# Prognostic value of flow-status in severe aortic stenosis patients undergoing percutaneous intervention

**DOI:** 10.1007/s10554-023-02992-x

**Published:** 2023-11-19

**Authors:** Diogo Santos-Ferreira, Isabel Fernandes, Sílvia O. Diaz, Cláudio Guerreiro, Francisca Saraiva, António S. Barros, Adelino Leite-Moreira, Eulália Pereira, Francisco Sampaio, José Ribeiro, Pedro Braga, Ricardo Fontes-Carvalho

**Affiliations:** 1 Cardiology Department, Centro Hospitalar de Vila Nova de Gaia/Espinho, Rua Conceição Fernandes, Vila Nova de Gaia, 4434-502 Portugal; 2 Department of Surgery and Physiology, UnIC@RISE, Faculty of Medicine, University of Porto, Alameda Prof. Hernâni Monteiro, Porto, 4200-319 Portugal

**Keywords:** Aortic valve stenosis, Flow rate, Low flow, Prognosis, Stroke volume index, Transcatheter aortic valve replacement

## Abstract

**Purpose:**

Low-flow status is a mortality predictor in severe aortic stenosis (SAS) patients, including after transcatheter aortic valve implantation (TAVI) treatment. However, the best parameter to assess flow is unknown. Recent studies suggest that transaortic flow rate (FR) is superior to currently used stroke volume index (SVi) in defining low-flow states. Therefore, we aimed to evaluate the prognostic value of FR and SVi in patients undergoing TAVI.

**Methods:**

A single-centre retrospective analysis of all consecutive patients treated with TAVI for SAS between 2011 and 2019 was conducted. Low-FR was defined as < 200 mL/s and low-SVi as < 35 mL/m^2^. Primary endpoint was all-cause five-year mortality, analyzed using Kaplan-Meier curves and Cox regression models. Secondary endpoint was variation of NYHA functional class six months after procedure. Patients were further stratified according to ejection fraction (EF < 50%).

**Results:**

Of 489 cases, 59.5% were low-FR, and 43.1% low-SVi. Low-flow patients had superior surgical risk, worse renal function, and had a higher prevalence of coronary artery disease. Low-FR was associated with mortality (hazard ratio 1.36, *p* = 0.041), but not after adjustment to EuroSCORE II. Normal-SVi was not associated with survival, despite a significative *p*-trend for its continuous value. No associations were found for flow-status and NYHA recovery. When stratifying according to preserved and reduced EF, both FR and SVi did not predict all-cause mortality.

**Conclusion:**

In patients with SAS undergoing TAVI, a low-FR state was associated with higher mortality, as well as SVi, but not at a 35 mL/m^2^ cut off.

**Supplementary Information:**

The online version contains supplementary material available at 10.1007/s10554-023-02992-x.

## Introduction

Aortic stenosis (AS) is the most common valvular heart disease in Europe and North America [1]. Its prevalence in the elderly is growing exponentially, with high impact in patients’ morbidity and mortality [1, 2].

Conventionally, the classification of a severe AS relies on the aortic valve area (AVA), mean transaortic gradient, and peak jet velocity [3], which are flow-dependent parameters [4, 5]. Not infrequently, this definition may be challenging, as up to a third of patients present discordant severity measures [6], requiring the use of additional parameters, including flow-state.

For the evaluation of flow in AS patients, the mostly used parameter is stroke volume index (SVi). It emerged given the discordant AS grading in patients with preserved left ventricle ejection fraction (LVEF) [7]. Low-flow is defined as a SVi < 35 mL/m^2^ [8]. Low-SVi has been associated with worse prognosis, as it was found to be an independent predictor of mortality in patients with AS, including after transcatheter aortic valve implantation (TAVI) [7, 9, 10]. However, it is not a strict measure of flow, but rather a volumetric one. Therefore, several studies have questioned whether SVi is the best parameter for aortic valve flow assessment [11].

In this context, transaortic flow rate (FR) has been suggested as a more appropriate and discriminative parameter to directly evaluate flow [12], since it incorporates the underlying information of both volume and time. Also, FR is not normalized to body surface area (BSA) and heart rate, and has the additional advantage of reflecting aortic valve resistance [6]. Recent evidence highlighted the prognostic value of FR over SVi [13, 14], with a FR below 200 mL/s being independently associated with mortality following aortic valve intervention [15]. Yet, there are still some disparities in these conclusions, and further studies are still required to draw more robust conclusions [16].

In this study we aimed to evaluate the prognostic value of low-FR status vs. low-SVi in severe AS patients treated with TAVI.

## Materials and methods

### Study design and group definition

A retrospective analysis of all consecutive TAVI procedures for severe AS performed in Centro Hospitalar Vila Nova de Gaia/Espinho between January 2011 and December 2019 was performed. All patients with pre-intervention echocardiographic data from our center were included. Patients were stratified according to flow status at baseline as low-FR (< 200 mL/s) or normal-FR (≥ 200 mL/s), and according to SVi as low-SVi (< 35 mL/m^2^) or normal-SVi (≥ 35 mL/m^2^).

### Ethics approval

This study was approved by local Ethics Committee and informed consent was waived due to the retrospective design of the study.

### Data collection

Clinical data included sex, age, body mass index (BMI), BSA, New York Heart Association (NYHA) functional class, alongside other cardiovascular comorbidities. Surgical risk was estimated through the European System for Cardiac Operative Risk Evaluation II (EuroSCORE II) score [17]. The risk of mortality and morbidity for aortic intervention was calculated using the Society of Thoracic Surgeons (STS) score [18].

**Fig. 1:**
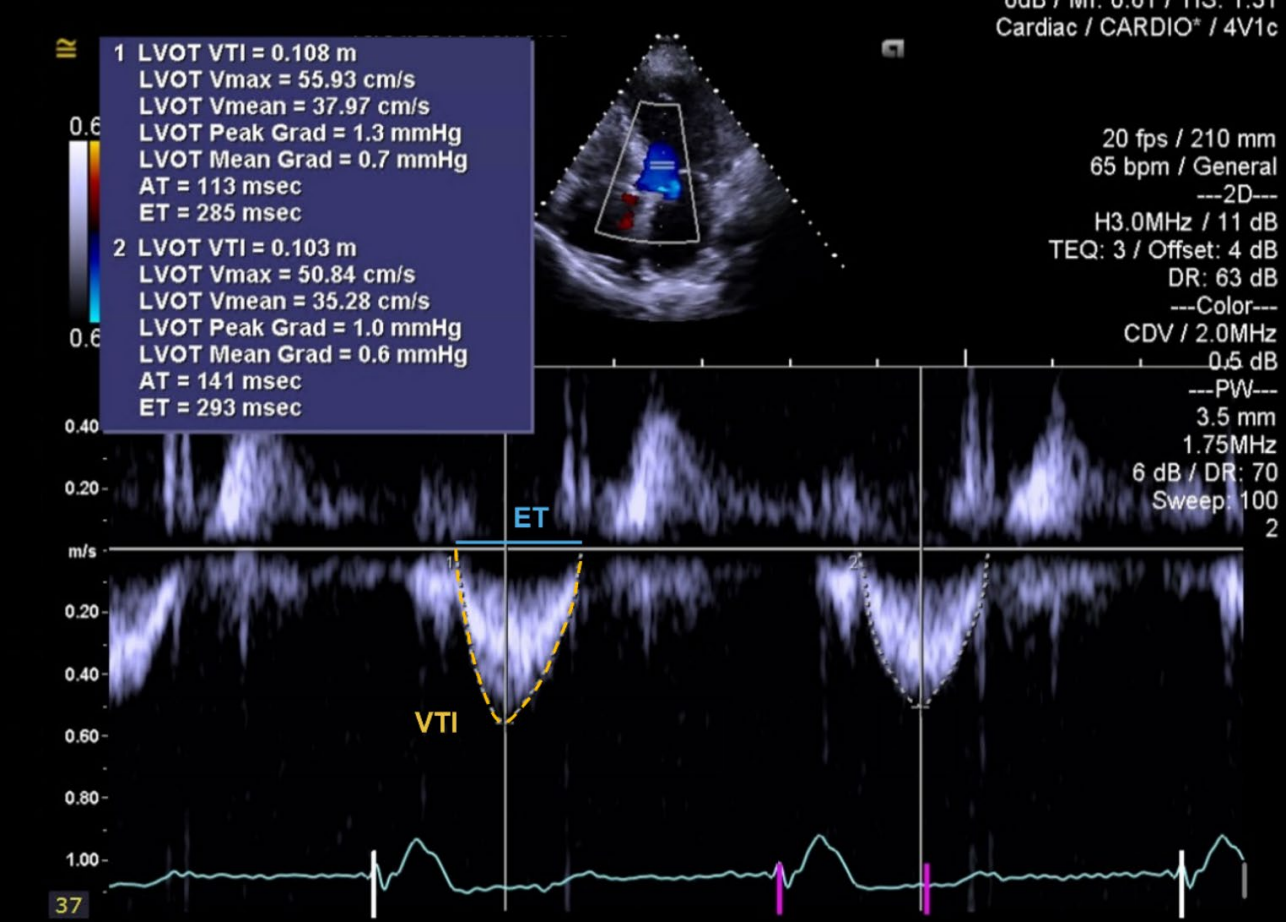
Pulse-wave Doppler in the left ventricle outflow tract (LVOT) of a patient with severe aortic stenosis. Transaortic flow rate (FR) is calculated dividing the Doppler-derived stroke volume by the ejection time (ET, in blue), using velocity time integral (VTI, in yellow) of the LVOT and respective LVOT diameter (LVOTd, not shown)

Doppler echocardiographic data included functional AVA, using the continuity equation), transaortic mean and maximum gradient, LVEF and SVi. Transaortic FR was prospectively calculated, using the available Doppler images, as the ratio of Doppler-derived stroke volume to systolic ejection time, using measured left ventricular outflow tract diameter and velocity time integral (Fig. 1), as described elsewhere [5]. FR indexed to BSA (FRi) was also determined. SVi was derived as the ratio of Doppler-derived stroke volume to BSA. These measurements were acquired blindly to patients’ characteristics and outcomes.

$$FR=\frac{VTI* \pi*{(\text{L}\text{V}\text{O}\text{T}\text{d}/2)}^{2} }{ET}$$

Follow-up data was obtained using health registry for mortality and hospital registries for NYHA class.

### Study endpoints

The primary endpoint was defined as all-cause mortality within five years after procedure. The secondary endpoint was NYHA functional class variation at six months after TAVI.

A subsequent analysis was performed further dichotomizing patients according to their ejection fraction (EF) before intervention, in reduced-EF (EF < 50%) vs. preserved-EF (EF ≥ 50%).

### Statistical analysis

Categorical variables are expressed as absolute values and percentages, and continuous variables are expressed as median, 25th and 75th percentiles (i.e., Q1 -Q3). Patients’ characteristics were compared between groups using χ^2^, Fisher’s or Wilcoxon tests, as appropriate. Statistical significance was considered at *p* < 0.05.

The primary endpoint analysis was performed using Kaplan-Meier curves (up to five-years) and log-rank test. Cox proportional hazard model was also employed to address the effect of low-FR or low-SVi on five-year mortality, univariately and adjusted for EuroSCORE II - a multiparametric risk score based on clinical, analytical, and echocardiographic data. Hazard Ratios (HR) with 95% confidence intervals and *p*-values were reported.

A *p*-trend was also calculated using the same Cox proportional hazard models considering the continuous values of FR, FRi and SVi. Proportional hazard assumption of the Cox models was tested using Schoenfeld residuals.

For the secondary endpoint, a NYHA functional class recovery ≥ 1 and ≥ 2 after six months, compared to pre-TAVI NYHA class, was evaluated.

All statistical analysis and plots were done using R statistical software, version 4.1.2. [19–22].

## Results

### Baseline characteristics

**Fig. 2:**
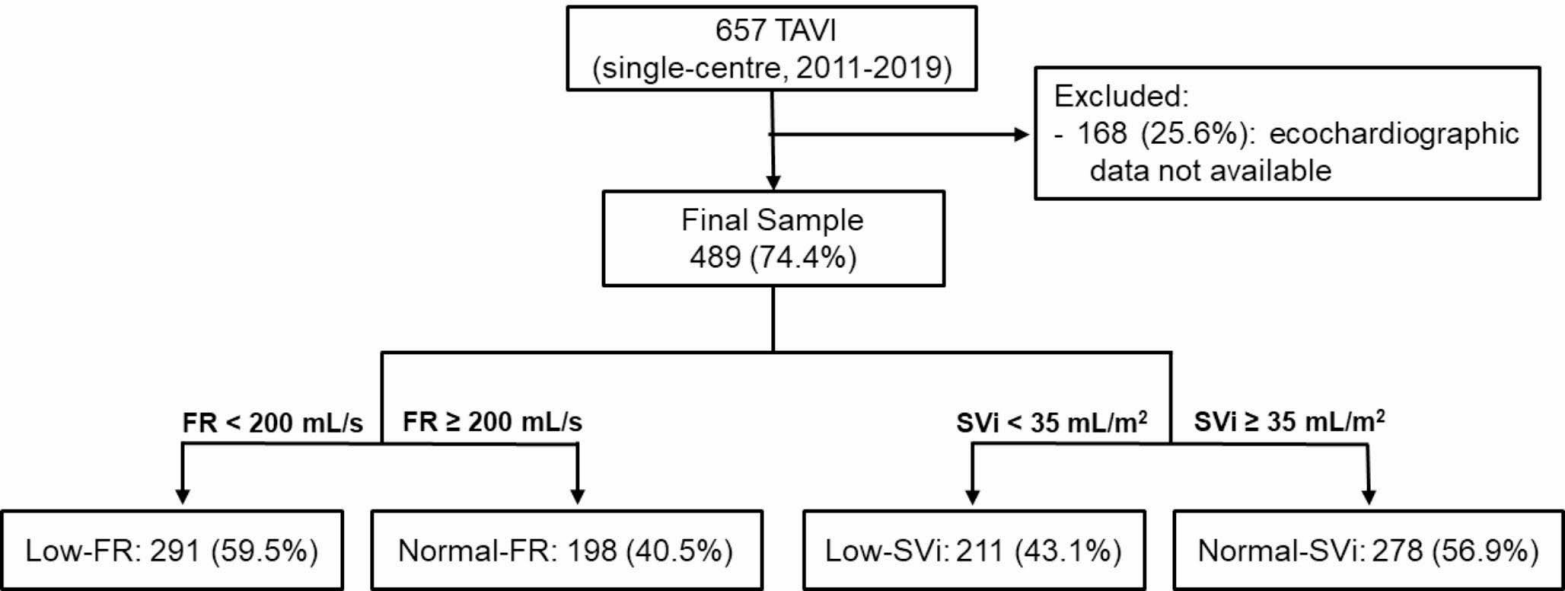
Flow chart of the study population. FR, flow rate; SVi, stroke volume index; TAVI, transcatheter aortic valve intervention

Among the 657 patients who underwent TAVI during the defined period, 489 (74.4%) had pre-intervention echocardiogram available, allowing the collection of FR and SVi values, as detailed in the algorithm in Fig. 2. Patient demographics and baseline characteristics are summarized in Table 1. This population was predominantly old (median age 81 years-old), with several comorbidities. More than half had coronary artery disease, whereas a third had atrial fibrillation (AF). Anemia was also prevalent in this subset (43%). Surgical risk was estimated to be low-to-intermediate, with a median EuroSCORE II of 4.1, and a median STS score for mortality of 4.0%. Most patients presented either in a NYHA class of II (43%) or III (49%) at baseline.

**Table 1:** Characteristics of the population included, divided according to flow rate and stroke volume index

Characteristic	Overall	FR < 200 mL/s (n = 291)	FR ≥ 200 mL/s, (n = 198)	*p* ^1^	SVi < 35 mL/m^2^, (n = 211)	SVi ≥ 35 mL/m^2^, (n = 278)	*p* ^1^
**Demographic and Clinical Data**							
Sex				**< 0.001**			0.2
Male	236 (48%)	116 (40%)	120 (61%)		94 (45%)	142 (51%)	
Female	253 (52%)	175 (60%)	78 (39%)		117 (55%)	136 (49%)	
Age (years)	81 [76, 86]	82 [78, 86]	80 [75, 84]	**< 0.001**	82 [78, 86]	81 [76, 85]	0.15
Body mass index (kg/m^2^)	26.8 [24.2, 29.8]	26.0 [23.6, 29.3]	27.7 [25.3, 30.5]	**< 0.001**	27.0 [24.3, 30.4]	26.7 [24.2, 29.4]	0.4
Body surface area (m^2^)	1.75 [1.64, 1.87]	1.70 [1.60, 1.83]	1.82 [1.71, 1.93]	**< 0.001**	1.75 [1.64, 1.87]	1.76 [1.63, 1.88]	0.8
Creatinine clearance (mL/m^2^)	48 [35, 64]	45 [33, 58]	53 [40, 72]	**0.001**	45 [34, 59]	51 [38, 66]	**0.028**
NYHA class ≥ III (baseline)	263 (56%)	168 (60%)	95 (50%)	**0.025**	130 (64%)	133 (50%)	**0.001**
NYHA class ≥ III (6-months follow-up)	29 (8.0%)	19 (9.0%)	10 (6.6%)	0.4	12 (8.3%)	17 (7.8%)	0.9
NYHA recovery ≥ 1	237 (66%)	97 (65%)	140 (67%)	0.7	137 (64%)	100 (70%)	0.2
NYHA recovery ≥ 2	66 (18%)	21 (14%)	45 (22%)	0.070	38 (18%)	28 (20%)	0.7
Arterial hypertension	360 (83%)	212 (83%)	148 (84%)	0.8	157 (84%)	203 (82%)	0.5
Diabetes mellitus	185 (38%)	105 (36%)	80 (41%)	0.3	90 (43%)	95 (34%)	**0.049**
Dyslipidemia	314 (73%)	183 (71%)	131 (74%)	0.6	136 (73%)	178 (72%)	0.8
COPD	92 (19%)	48 (17%)	44 (22%)	0.11	36 (17%)	56 (20%)	0.4
Carotid artery disease	57 (12%)	38 (13%)	20 (10%)	0.6	26 (12%)	32 (11%)	0.9
Previous stroke or TIA	63 (13%)	42 (15%)	21 (11%)	0.2	30 (14%)	33 (12%)	0.4
Peripheral vascular disease	48 (11%)	32 (12%)	16 (9.0%)	0.3	23 (12%)	25 (10%)	0.5
Coronary artery disease	267 (55%)	171 (59%)	96 (49%)	**0.023**	131 (63%)	136 (49%)	**0.003**
Previous PCI	77 (16%)	56 (19%)	21 (11%)	**0.010**	45 (22%)	32 (12%)	**0.003**
Previous CABG	67 (14%)	44 (15%)	23 (12%)	0.3	32 (15%)	35 (13%)	0.4
Previous pacemaker	59 (14%)	33 (13%)	26 (15%)	0.5	29 (15%)	30 (12%)	0.3
Atrial fibrillation	160 (33%)	99 (34%)	61 (31%)	0.4	88 (42%)	72 (26%)	**< 0.001**
Anemia	187 (43%)	116 (45%)	71 (40%)	0.3	83 (45%)	104 (42%)	0.6
STS score (mortality, %)	4.00 [2.76, 5.98]	4.37 [3.03, 6.63]	3.47 [2.46, 5.04]	**< 0.001**	4.34 [2.93, 6.98]	3.82 [2.60, 5.48]	**0.003**
STS score (morbimortality, %)	22 [17, 29]	23 [18, 31]	20 [14, 26]	**< 0.001**	24 [18, 31]	21 [15, 27]	**0.003**
EuroSCORE II	4.1 [2.4, 6.5]	4.5 [3.0, 7.3]	3.4 [1.9, 5.4]	**< 0.001**	4.8 [3.0, 8.4]	3.7 [2.1, 5.5]	**< 0.001**
Five-year mortality (%)	194 (40%)	130 (45%)	67 (34%)	**0.016**	94 (45%)	103 (37%)	0.094
**Echocardiographic data**							
Aortic valve area (cm^2^)	0.67 [0.50, 0.80]	0.60 [0.50, 0.70]	0.70 [0.60, 0.80]	**< 0.001**	0.60 [0.50, 0.70]	0.70 [0.60, 0.80]	**< 0.001**
Transaortic maximum gradient (mmHg)	76 [63, 92]	75 [62, 92]	79 [66, 92]	0.10	71 [58, 86]	79 [68, 95]	**< 0.001**
Transaortic mean gradient (mmHg)	46 [39, 57]	45 [38, 55]	47 [40, 58]	0.053	43 [35, 53]	48 [41, 60]	**< 0.001**
Ejection fraction (%)	55 [45, 59]	55 [44, 58]	55 [50, 60]	**0.026**	54 [40, 57]	56 [51, 60]	**< 0.001**
Ejection fraction < 50%	140 (29%)	93 (32%)	47 (24%)	**0.048**	79 (37%)	61 (22%)	**< 0.001**
Stroke volume index (mL/m^2^)	37 [30, 44]	31 [26, 37]	45 [41, 52]	**< 0.001**	29 [25, 32]	42 [39, 49]	**< 0.001**
Transaortic flow rate (mL/s)	190 [158, 230]	164 [141, 183]	240 [217, 266]	**< 0.001**	155 [132, 176]	217 [194, 255]	**< 0.001**

^1^Pearson’s Chi-squared test; Wilcoxon rank sum test; Fisher’s exact test

CABG, Coronary artery bypass graft; COPD, chronic obstructive pulmonary disease; FR, Flow rate; NYHA, New York Heart Association; PCI, Percutaneous Coronary Intervention; STS, Society of Thoracic Surgeons; SVi, Stroke Volume Index; TIA, transient ischemic attack. Statistically significant *p*-values are presented in bold

In this population, 59.5% were considered low-flow according to FR, and 43.1% were low-flow according to SVi (Table 2). Patients’ classification as low- or normal-flow using each parameter was concordant in 77.5% of patients, with 40.1% of patients having low-FR and low-SVi, and 37.4% having normal-FR and normal-SVi. For the remaining patients, 3.1% had normal-FR but low-SVi and 19.4% normal-SVi and low-FR. There was a statistically significant positive Pearson’s correlation in flow classification according to FR and SVi (r = 0.59, *p* < 0.001).

**Table 2:** Flow status distribution, according to flow rate and stroke volume index

	SVi < 35 mL/m^2^	SVi ≥ 35 mL/m^2^	Total
FR < 200 mL/s	196 (40,1%)	95 (19,4%)	291 (59.5%)
FR ≥ 200 mL/s	15 (3,1%)	183 (37,4%)	198 (40.5%)
Total	211 (43.1%)	278 (56.9%)	489

FR, Flow rate; SVi, Stroke volume index

Low-flow patients, defined by either FR and SVi, had higher estimated surgical risk (EuroSCORE II and STS scores) and were in more advanced NYHA classes. Those patients also had a lower estimated creatinine clearance and had more frequently coronary artery disease, including previous percutaneous coronary intervention. In the low-FR group, there was a higher predominance of female sex (60% vs. 39%, *p* < 0.001) and slightly older patients (82- vs. 80-years-old, *p* < 0.001), with lower BMI and BSA. Regarding comorbidities, diabetes mellitus and AF was more prevalent among low-SVi patients versus normal-SVi.

Regarding echocardiographic data, both low-FR and low-SVi groups had lower functional AVA when compared to normal-FR and normal-SVi patients (0.60 vs. 0.70 cm^2^, *p* < 0.001), as well as a lower EF, with a higher predominance of reduced-EF patients. In addition, low-SVi, but not low-FR, was associated with lower transaortic gradients before TAVI.

### Endpoints

Median follow-up after TAVI was 46 months [Q1 33, Q3 65]. 40% of patients died within five years after TAVI.

**Fig. 3:**
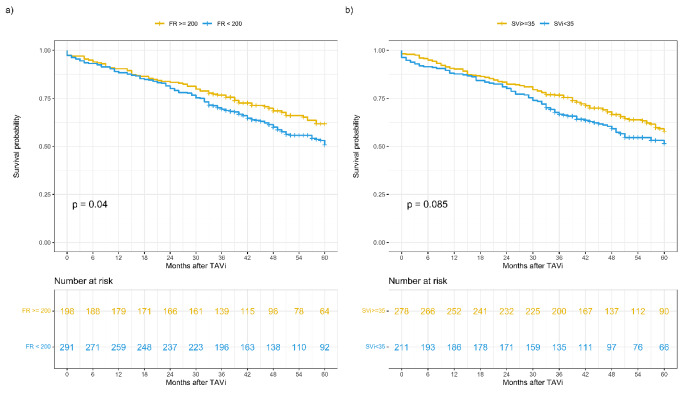
Kaplan-Meier survival curves for five-year all-cause mortality, according to (A) flow rate (FR) and (B) stroke volume index (SVi) before transcatheter aortic valve intervention

Patients with low-FR exhibited a lower five-year survival after TAVI than patients with normal-FR (55% vs. 66%, *p* = 0.04). No statistically significant differences were found regarding mortality within five years after valvular intervention between low-SVi and normal-SVi patients (55% vs. 63%, *p* = 0.085) (Fig. 3).

A low-FR was associated with a higher all-cause mortality over the follow-up period in the univariable Cox regression [Hazard Ratio (HR): 1.36; 95%CI: 1.01 to 1.83; *p* = 0.041], but not after adjustment for EuroSCORE II (Table 3). A low-SVi was not associated with a worse prognosis after intervention. When considering FR and SVi as continuous variables, higher SVi, but not FR, was associated with lower mortality (*p*-trend 0.031 and 0.095, respectively) but not after adjustment to EuroSCORE II (Table 3). There was a marginally non-significant association of FRi and the primary endpoint, presented per 10 mL/m^2^s increase in the supplemental Table 1 (HR: 0.96, 95%CI: 0.91-1.00, *p* = 0.061).

**Table 3:** Univariable and Multivariable Cox Regression for categorical flow rate and stroke volume index

Variable	Univariable Analysis			Multivariable Analysis^1^		
	Hazard Ratio (95% CI)	*p*-value	*p*-trend	Hazard Ratio (95% CI)	*p*-value	*p*-trend
Low-FR	1.36 (1.01, 1.83)	**0.041**	0.095	1.33 (0.98, 1.81)	0.065	0.2
Low-SVi	1.28 (0.97, 1.69)	0.085	**0.031**	1.20 (0.89, 1.61)	0.2	0.069
**Preserved EF**						
Low-FR	1.33 (0.94, 1.89)	0.11	0.2	1.34 (0.93, 1.92)	0.12	0.2
Low-SVi	1.16 (0.82, 1.64)	0.4	0.12	1.09 (0.76, 1.56)	0.6	0.2
**Reduced EF**						
Low-FR	1.37 (0.78, 2.39)	0.3	0.3	1.37 (0.77, 2.45)	0.3	0.5
Low-SVi	1.48 (0.88, 2.48)	0.13	0.2	1.44 (0.85, 2.45)	0.2	0.2

^1^Adjusted to EuroSCORE II

CI: Confidence Interval; EF, Ejection fraction; FR, Flow rate; SVi, Stroke volume index. Statistically significant *p*-values are presented in bold

Regarding the survival analysis according to LVEF, low-FR or low-SVi states were not associated with mortality within the five years after TAVI in both patients with normal-EF or reduced-EF (Table 3).

Baseline NYHA class was more advanced among low-FR (patients in NYHA ≥ III 60.2% vs. 49.7%) and low-SVi patients (patients in NYHA ≥ III 64.4% vs. 49.6%). There was an improvement in NYHA functional assessment, with a recovery of one or more classes at six months after intervention in about two-thirds of the patients, which was no different for normal or low-flow patients, either given by FR or SVi (Table 1). There was a trend towards a more frequent recovery of NYHA class ≥ 2 among normal-FR vs. low-FR patients although not statistically significant (22% vs. 14%, *p* = 0.07).

Despite exhibiting more advanced pre-TAVI NYHA classes, low-flow patients had a similar NYHA class at six-months follow-up when compared to normal-flow counterparts, either considering FR (9% vs. 6.6% of patients had NYHA ≥ III) or SVi (8.3% vs. 7.8% of patients presented NYHA ≥ III).

## Discussion

The impact of a low-flow state in severe AS patients treated with TAVI, either considering FR or SVi, was assessed and compared. The key findings of the present study are that: (1) a relevant proportion of patients with severe AS have low transvalvular flow states, either assessed using FR or SVi; (2) low-FR is associated with higher all-cause mortality post-TAVI; (3) SVi is associated with mortality after intervention, but not when dichotomized at < 35 mL/m^2^; (4) low-flow patterns are associated with clinical characteristics portraying worse outcomes and a higher estimated risk, and are not independently associated with worse survival.

### The low-flow profile

In the present analysis of 489 patients undergoing TAVI, 62.6% presented low-SVi and/or low-FR, denoting that this low-flow status is highly prevalent in a “real-world” cohort of severe AS patients undergoing percutaneous treatment. Thus, further characterization, including the impact of specific management strategies, should be specifically addressed in this subset. In other severe AS cohorts, low-flow state, considering either SVi or FR, was found in 55% of the patients included [23, 24], reinforcing that this condition is not epidemiologically negligible.

Our results show that low-flow severe AS patients are of higher estimated surgical risk, and present more comorbidities, including coronary heart disease and previous percutaneous coronary intervention. Furthermore, a low-flow state is associated with aggravated symptoms of heart failure, based on NYHA classification.

### Stroke volume index and flow rate – the same, only different?

There is uncertainty about which measure is the most suitable for assessing flow through the aortic valve, though the superiority of FR as a flow measure has been suggested [25, 26]. In a sub-analysis of the SEAS (Simvastatin and Ezetimibe in Aortic Stenosis) population, 21% of patients had low transaortic FR at baseline, but only 10% had low-SVi [13]. In another severe AS cohort, 50% of patients exhibited a low-SVi, but only 39% had low-FR [23]. In our population, 59.5% of patients had a low transaortic FR at baseline, but only 43.1% had a low SVi. As both measures are stroke volume dependent, it is expected that these are associated, and our results confirm a moderate positive correlation between them. However, almost one-fourth of patients presented discording flow-states if defined by FR or SVi, being low-FR but normal SVi the most frequent discordance (19.4%). This can be partly explained by the SVi dependence on BSA, contrary to FR [6]. Another potential source for discordance is the higher dependence of SVi on heart rate – patients with higher rates have lower left ventricular ejection time and thus may present with lower SVi despite normal FR [24].

The present study found an association between low-FR and female sex, older age, and lower BMI and BSA, which is in agreement with previous reports [7, 27, 28]. There are several causes pointed for FR (but not SVi) being lower among women, including a lower stroke volume (unindexed) [28], being the latter corrected to a tendentially lower BSA in female patients, which is not currently applicable for FR. On the other hand, AF was more frequent among low-SVi (but not low-FR) cases, which can be integrated in a context of no atrial systolic contribution to left ventricular filling, as well as impaired filling time due to poor ventricular rate control, as described in the literature [29].

A reduced transvalvular flow has recently been associated with a worse systolic function [7], and in the present study both low-FR and low-SVi patients exhibited a lower LVEF, and a higher frequency of low-EF cases. Furthermore, Baron S. et al. reported that ventricular dysfunction was not independently associated with one-year mortality after TAVI [30], suggesting that the prognostic value of FR and SVi is not related to differences in ventricular dysfunction.

Low-flow states can be associated with low transvalvular gradients and erroneously low AVA, since its true measurement is dependent on achieving sufficient transaortic flow to maximize the leaflets opening [5]. In the present analysis, AVA was lower in low-flow patients, according to FR or SVi definition, and low-SVi – but not low-FR - patients exhibited lower transaortic gradients, when compared to normal-SVi.

The role of assessing FR in patients with low-flow, low-gradient AS, and the added value of dobutamine stress echocardiography (SE) has been studied. Chahal et al. found that in patients with low-flow, low-gradient AS, a resting FR, but not SVi or LVEF, predicted AVA changes during SE, and a resting AVA measured under normal-FR state truly reflected AS severity, potentially obviating the need of SE [26]. In patients presenting a low SVi and low AVA, a normal FR suggests that the valvular opening forces are normal and that AS is truly severe, contrary to a potential underestimation of AVA in the context of a low FR [24].

### The prognostic value of low-flow and how it should be managed

There was an association between low-FR and a 36% higher mortality in patients undergoing TAVI for severe AS. This was consistent with findings from another study on patients proposed for aortic valve intervention, including valvuloplasty and surgical or transcatheter replacement, in which a low-FR had HR = 2.95 for all-cause mortality after procedure, even after adjusting to other factors, including EuroSCORE II [15]. In the present study, there was a tendency towards a higher mortality after TAVI in low-FR patients when corrected to EuroSCORE II, though not reaching statistical significance (*p* = 0.065). Additionally, a low-FR seems to be associated with higher mortality across different AS states, as this was also seen in initially asymptomatic mild to moderate AS patients [13].

Regarding the prognostic value of SVi in severe AS patients proposed for TAVI, our results denoted that there was a trend towards reduced five-year survival of patients with SVi < 35 mL/m^2^ versus normal-SVi counterparts, though it did not reach statistical significance (*p* = 0.085). In fact, a low-SVi has been consistently associated with a higher mortality after TAVI – according to a meta-analysis, there is a 59% increase in one-year mortality after intervention [10]. It can be hypothesized that these findings were not replicated in the present study possibly due to a relatively low number of patients included. Nevertheless, in the present study, when considering SVi as a continuous variable, a higher SVi was associated with a reduction in mortality after TAVI. Therefore, we speculate that, in our population, there is an association between SVi and survival after intervention, but not when dichotomized at a < 35 mL/m^2^ threshold. Concerns have been raised regarding the optimal cut off to prognostically-define a low-SVi patient, and if it should vary according to sex – namely 40 mL/m^2^ and 32 mL/m^2^ for men and women, respectively, according to a study in surgically-treated aortic stenosis patients [31].

The prognostic performance of low-FR and low-SVi has been previously compared. Both are associated with worse outcomes in severe AS patients [16], including low-gradient subset [23]. While Alexandru et al. concluded that both had similar accuracy in predicting death, Sen et al. found that SVi, but not FR, improve risk reclassification when compared with clinical and echocardiographic predictors in low-gradient severe AS patients for a composite endpoint of death and heart failure hospitalization [23].

There are some controversies regarding the additive prognostic role of FR and SVi. In normal-SVi patients, a low-FR is associated with worse prognosis in moderate and severe AS patients [27], but this was not seen in low-gradient severe AS patients who underwent aortic valve procedures [15]. In low-SVi patients, a low-FR predicted higher mortality after valvular intervention [15]. On the other hand, SVi has an impact on mortality on low-FR and normal-FR low-gradient severe AS patients [23], but did not impact survival among treated-AS normal-FR and low-FR patients [15].

Given the difficulty in managing low-flow patients, some studies have suggested that treating them by valvular intervention rather than medically may result in a better prognosis [7]. Saeed et al. reported that all-cause mortality was significantly lower in the aortic valve intervention group (including surgical, transcatheter and balloon aortic valvuloplasty) compared with standard medical treatment, both when low-flow was defined by FR (13.6 vs. 52.3%, *p* < 0.001) and SVi (15.1 vs. 45.9%, *p* < 0.001) [32]. Another study demonstrated the superiority of valvular intervention over medical treatment, with TAVI reducing mortality in low-flow patients (HR: 0.48, *p* = 0.004) [9]. The present study reveals that, despite being associated with worse survival after intervention, the relationship between low-flow states and mortality seems to be related to an underlying clinical context of comorbidities and more advanced cardiac disease, as the association lost statistical significance when adjusted to EuroSCORE II. Therefore, a low-flow condition should not, *per se*, condition the decision for percutaneous intervention, but alternatively be integrated in an appropriate procedural risk assessment.

Discordant values of AS assessments are stated to occur both at preserved and reduced LVEF [33]. In our population, LVEF was lower and more frequently reduced in both low-FR and low-SVi patients. When reclassifying our population according to this parameter in preserved- *versus* reduced-EF at a 50% cut off, neither flow parameter retained any prognostic value.

Nevertheless, in the present study, there was a clear symptomatic benefit from performing TAVI in these severe AS patients, with a reduction in heart failure symptoms, evaluated through a substantial functional improvement of NYHA class at six-month, irrespectively of flow state. Thus, a similar symptomatic improvement might be expected in treated low-flow *versus* normal-flow patients. Given the poor prognosis of severe AS medically treated and the clear symptomatic benefits of TAVI in these patients, this procedure should be considered as a viable treatment option in patients with low-flow AS, whether assumed using FR or SVi.

### Transaortic flow rate – where should we draw the line?

The most adequate cut-off defining low-flow according to FR remains conflicting, with studies using a wide variety of cut-offs, ranging from 200 to 250 mL/s. Namasivayam et al. referred to “low-FR” as below the median found in the respective study population (242 mL/s) [27], while other groups found 211 mL/s as the best cut-off value for predicting death from all-causes [14]. The first reported cut-off of 200 mL/s was described in an in vitro experience, considering a normal cardiac output of 5 L/min [34]. As the cut-off of 200 mL/s was the most reported in the literature and for which there was stronger evidence [13, 15, 24–26, 34], it was the one considered for the present analysis.

### Limitations and strengths

The present study is a retrospective single-center analysis, and marginally non-significant findings might have been conditioned by an insufficient number of patients included. About 25% of patients undergoing TAVI were excluded due to incomplete echocardiographic data, potentially representing a selection bias. As our population was limited to cases with severe AS subjected to TAVI, no extrapolations of this data should be taken for medically or surgically treated low-flow patients. Also, echocardiographic measurements are severely operator-dependent, with an inherent random variability of the collected data, registered blindly to the endpoints defined. Additional information about the cause of death (cardiovascular vs. non-cardiovascular) was not available, as well as other endpoints, such as hospitalizations during follow-up, and therefore no further conclusions could be drawn.

This study is one of the few that questions whether FR represents a mortality predictor in patients who underwent percutaneous aortic valve intervention, and further details on the role of assessing SVi before TAVI as a prognostic marker.

## Conclusions

In severe AS patients undergoing TAVI, low-flow states are common and associated with more advanced symptoms and higher estimated procedural risk. A low-FR negatively impacts survival over the five-years after intervention. SVi is also associated with mortality, but not at a < 35 mL/m^2^ cut off, which merits a further investigation on optimal low-SVi definition regarding prognosis in this relevant subset of patients.

### Clinical implications

The present study strengthens the well-established need for a more comprehensive evaluation of AS severity beyond classic measurements. Our results suggest that the pre-procedural flow-states have an important prognostic value for all-cause mortality after TAVI, emphasizing the role of these parameters in the current evaluation and risk assessment of patients with severe AS.

### Electronic supplementary material

Below is the link to the electronic supplementary material.

Supplementary Material 1

## References

[1] Osnabrugge RL, Mylotte D, Head SJ (2013). Aortic stenosis in the elderly: Disease prevalence and number of candidates for transcatheter aortic valve replacement: a meta-analysis and modeling study. J Am Coll Cardiol.

[2] Eveborn GW, Schirmer H, Heggelund G (2013). The evolving epidemiology of valvular aortic stenosis. The Tromsø study. Heart.

[3] Vahanian A, Beyersdorf F, Praz F (2021). 2021 ESC/EACTS guidelines for the management of valvular Heart Disease. Eur J Cardiothorac Surg.

[4] Sherwood MW, Kiefer TL (2017). Challenges in aortic valve stenosis: low-Flow States diagnosis, management, and a review of the current literature. Curr Cardiol Rep.

[5] Burwash IG, Thomas DD, Sadahiro M (1994). Dependence of Gorlin formula and continuity equation valve areas on transvalvular volume flow rate in valvular aortic stenosis. Circulation.

[6] Lazaros G, Drakopoulou MI, Tousoulis D (2018). Transaortic Flow in aortic stenosis: Stroke volume Index versus Flow Rate. Cardiology.

[7] Hachicha Z, Dumesnil JG, Bogaty P (2007). Paradoxical low-flow, low-gradient severe aortic stenosis despite preserved ejection fraction is associated with higher afterload and reduced survival. Circulation.

[8] Baumgartner HC, Hung JC-C, Bermejo J (2017). Recommendations on the echocardiographic assessment of aortic valve stenosis: a focused update from the European Association of Cardiovascular Imaging and the American Society of Echocardiography. Eur Heart J Cardiovasc Imaging.

[9] Herrmann HC, Pibarot P, Hueter I (2013). Predictors of mortality and outcomes of therapy in low-flow severe aortic stenosis: a Placement of aortic transcatheter valves (PARTNER) trial analysis. Circulation.

[10] Eleid MF, Goel K, Murad MH (2015). Meta-analysis of the prognostic impact of Stroke volume, gradient, and Ejection Fraction after Transcatheter aortic valve implantation. Am J Cardiol.

[11] Bansal P, Maini A, Abbas A (2021). Transaortic Flow in aortic stenosis: Stroke volume Index versus Transaortic Flow Rate. J Am Soc Echocardiogr.

[12] Pibarot P, Dumesnil JG (2012). Low-flow, low-gradient aortic stenosis with normal and depressed left ventricular ejection fraction. J Am Coll Cardiol.

[13] Saeed S, Senior R, Chahal NS (2017). Lower Transaortic Flow rate is Associated with increased mortality in aortic valve stenosis. JACC Cardiovasc Imaging.

[14] Vamvakidou A, Jin W, Danylenko O (2019). Impact of Pre-intervention Transaortic Flow Rate Versus Stroke volume index on Mortality across the hemodynamic spectrum of severe aortic stenosis: implications for a new hemodynamic classification of aortic stenosis. JACC Cardiovasc Imaging.

[15] Vamvakidou A, Jin W, Danylenko O (2019). Low Transvalvular Flow Rate predicts mortality in patients with low-gradient aortic stenosis following aortic valve intervention. JACC Cardiovasc Imaging.

[16] Alexandru D, Pollack S, Petillo F (2018). The utility of Flow Rate compared with left ventricular Stroke volume index in the hemodynamic classification of severe aortic stenosis with preserved ejection fraction. Cardiology.

[17] Nashef SA, Roques F, Sharples LD (2012). EuroSCORE II. Eur J Cardiothorac Surg.

[18] O’Brien SM, Feng L, He X (2018). The Society of thoracic surgeons 2018 adult cardiac Surgery risk models: part 2-Statistical methods and results. Ann Thorac Surg.

[19] R Core Team (2021) R: A language and environment for statistical computing. R Foundation for Statistical Computing, Vienna, Austria. https://www.R-project.org/

[20] Kassambara A, Kosinski M, Biecek P (2021) survminer: Drawing Survival Curves using ‘ggplot2’. R package version 0.4.9. https://CRAN.R-project.org/package=survminer

[21] Therneau T (2023) A Package for Survival Analysis in R. R package version 3.5-5.: https://CRAN.R-project.org/package=survival

[22] Sjoberg DD, Whiting K, Curry M, Lavery JA, Larmarange J (2021). Reproducible summary tables with the gtsummary package. R J.

[23] Sen J, Huynh Q, Stub D (2021). Prognosis of severe Low-Flow, low-gradient aortic stenosis by Stroke volume index and Transvalvular Flow Rate. JACC Cardiovasc Imaging.

[24] Clavel MA, Annabi MS (2021). Low-Flow aortic stenosis: Flow Rate does not replace but could refine Stroke volume index. JACC Cardiovasc Imaging.

[25] Vamvakidou A, Chahal N, Senior R (2017). Lack of Stroke volume determined Flow Reserve does not always preclude Assessment of Severity of aortic stenosis in Low-Flow Low-Gradient State during Dobutamine Echocardiography. JACC Cardiovasc Imaging.

[26] Chahal NS, Drakopoulou M, Gonzalez-Gonzalez AM (2015). Resting aortic valve area at normal transaortic Flow Rate reflects true Valve Area in suspected low-gradient severe aortic stenosis. JACC Cardiovasc Imaging.

[27] Namasivayam M, He W, Churchill TW (2020). Transvalvular Flow Rate determines Prognostic Value of aortic valve area in aortic stenosis. J Am Coll Cardiol.

[28] Saeed S, Vamvakidou A, Zidros S (2021). Sex differences in transaortic flow rate and association with all-cause mortality in patients with severe aortic stenosis. Eur Heart J Cardiovasc Imaging.

[29] Leong DP, Pizzale S, Haroun MJ (2016). Factors Associated with Low Flow in aortic valve stenosis. J Am Soc Echocardiogr.

[30] Baron SJ, Arnold SV, Herrmann HC (2016). Impact of Ejection Fraction and aortic valve gradient on outcomes of transcatheter aortic valve replacement. J Am Coll Cardiol.

[31] Guzzetti E, Poulin A, Annabi MS (2020). Transvalvular Flow, Sex, and Survival after Valve replacement Surgery in patients with severe aortic stenosis. J Am Coll Cardiol.

[32] Saeed S, Vamvakidou A, Seifert R (2019). The impact of aortic valve replacement on survival in patients with normal flow low gradient severe aortic stenosis: a propensity-matched comparison. Eur Heart J Cardiovasc Imaging.

[33] Minners J, Allgeier M, Gohlke-Baerwolf C (2008). Inconsistencies of echocardiographic criteria for the grading of aortic valve stenosis. Eur Heart J.

[34] Voelker W, Reul H, Ing GN (1995). Comparison of Valvular Resistance, Stroke Work loss, and Gorlin Valve Area for quantification of aortic stenosis. Circulation.

