# Supplementary material for: Prognostic value of flow-status in severe aortic stenosis patients undergoing percutaneous intervention
Source: Int J Cardiovasc Imaging. 2023 Nov 19;40(2):341–50. doi: 10.1007/s10554-023-02992-x (PMC10884040; doi:10.1007/s10554-023-02992-x)
Supplement: Supplementary file 1 — Supplementary Material 1 [file 10554_2023_2992_MOESM1_ESM.docx]

**Supplementary table 1.** Univariable and Multivariable Cox Regression for flow rate indexed to body surface area.

| Variable |  |  | Univariable Analysis | | |  |  |  | Multivariable Analysis^1^ | | |
| --- | --- | --- | --- | --- | --- | --- | --- | --- | --- | --- | --- |
|  |  | Hazard Ratio (95% CI) | | *p*-value | |  |  | Hazard Ratio (95% CI) | | *p*-value | |
| Indexed FR (mL/m^2^s)/10 |  | 0.96 (0.91, 1.00) | | 0.061 | |  |  | 0.96 (0.92, 1.01) | | 0.10 | |
| **Preserved EF** |  |  | |  |  |  |  |  | |  |  |
| Indexed FR (mL/m2s)/10 |  | 0.96 (0.91, 1.01) | | 0.15 | |  |  | 0.96 (0.90, 1.02) | | 0.2 | |
| **Reduced EF** |  |  | |  |  |  |  |  | |  |  |
| Indexed FR (mL/m^2^s)/10 |  | 0.96 (0.88, 1.04) | | 0.3 | |  |  | 0.96 (0.89, 1.05) | | 0.4 | |

^1^Adjusted to EuroSCORE II

CI: Confidence Interval; EF, Ejection fraction; FR, Flow rate (results shown per 10mL/m^2^s variation)
